# Range modulation in proton therapy planning: a simple method for mitigating effects of increased relative biological effectiveness at the end-of-range of clinical proton beams

**DOI:** 10.1186/1748-717X-9-2

**Published:** 2014-01-02

**Authors:** Jeffrey C Buchsbaum, Mark W McDonald, Peter AS Johnstone, Ted Hoene, Marc Mendonca, Chee-Wei Cheng, Indra J Das, Kevin P McMullen, Mark R Wolanski

**Affiliations:** 1 Department of Radiation Oncology, Indiana University School of Medicine, Indianapolis, IN, USA; 2 IU Health Proton Therapy Center, Bloomington, IN, USA

**Keywords:** Proton therapy, Bragg peak, Toxicity, Proton dosimetry, Relative biological effectiveness (RBE), Patient safety, Treatment planning

## Abstract

**Background:**

The increase in relative biological effectiveness (RBE) of proton beams at the distal edge of the spread out Bragg peak (SOBP) is a well-known phenomenon that is difficult to quantify accurately *in vivo*. For purposes of treatment planning, disallowing the distal SOBP to fall within vulnerable tissues hampers sparing to the extent possible with proton beam therapy (PBT). We propose the distal RBE uncertainty may be straightforwardly mitigated with a technique we call “range modulation”. With range modulation, the distal falloff is smeared, reducing both the dose and average RBE over the terminal few millimeters of the SOBP.

**Methods:**

One patient plan was selected to serve as an example for direct comparison of image-guided radiotherapy plans using non-range modulation PBT (NRMPBT), and range-modulation PBT (RMPBT). An additional plan using RMPBT was created to represent a re-treatment scenario (RMPBTrt) using a vertex beam. Planning statistics regarding dose, volume of the planning targets, and color images of the plans are shown.

**Results:**

The three plans generated for this patient reveal that in all cases dosimetric and device manufacturing advantages are able to be achieved using RMPBT. Organ at risk (OAR) doses to critical structures such as the cochleae, optic apparatus, hypothalamus, and temporal lobes can be selectively spared using this method. Concerns about the location of the RBE that did significantly impact beam selection and treatment planning no longer have the same impact on the process, allowing these structures to be spared dose and subsequent associated issues.

**Conclusions:**

This present study has illustrated that RMPBT can improve OAR sparing while giving equivalent coverage to target volumes relative to traditional PBT methods while avoiding the increased RBE at the end of the beam. It has proven easy to design and implement and robust in our planning process. The method underscores the need to optimize treatment plans in PBT for both traditional energy dose in gray (Gy) and biologic dose (RBE).

## Background

Proton beam therapy (PBT) has emerged as an important advance in radiation therapy, particularly for children and young adults. Although there are data supporting equivalent rates of cure using equivalent doses of proton versus photon treatments, the decreased dose to nearby OARs and substantial reduction of irradiated tissue volume is a promising strategy to address acute and late injury to normal tissues from therapy [1]. Additionally, a matched pair analysis [2] supports a range from no difference to fewer second malignancies in patients treated with protons versus photon therapy. Similar data comparing photon CSI to proton CSI from multiple institutions also suggests a clear correlation of irradiated volume to risk of second malignancy [3-6].

Increased tissue effects at the end of the spread out Bragg Peak (SOBP) is a defined, measured biologic phenomenon [7-10]. The potential for unintentional tissue injury due to putative increased relative biological effectiveness (RBE) at the distal edge of a proton beam is an issue that must be addressed during the planning process [8,11-13]. The existing models suggest there may be a 5-10% increase in biological effect at the most distal portion of the SOBP relative to the plateau and an extension of effective proton range by 1–2 mm independent of fractionation and tissue type, while noting “There are no proton RBE values based on human-tissue response data, despite clinical experience of the treatment of more than 50,000 patients”. [ICRU-78 [14]; Section 2.4] Current areas of research are exploring how the RBE of protons also varies with fraction size and how it affects range [15,16].

In recognition of this dilemma, some current pediatric treatment protocols mandate multi-beam proton plans with the rationale that multiple beam entry directions will reduce the dose to critical structures in close proximity to the tumor. However, adding more beams may be detrimental in some cases or may simply increase logistics without therapeutic benefit. An example is the re-treatment of a brain tumor in which the brainstem, skin, and surrounding tissues have already acquired a significant dose from prior treatment. There may not be an optimal beam entry direction, let alone multiple entry directions in such cases. To address the issue of a potential increased RBE at the distal edge of a proton beam, another approach chooses to stop the beam beyond, rather than in, the OAR in order to place the distal portion of the SOBP in less vulnerable tissue downstream. This requires acceptance of the OAR receiving full dose uniformly as a safety measure in order to avoid a potentially serious, but poorly quantifiable, complication. Using mixtures of these methods, it is possible that OARs would receive more dose than they would from treatment with an alternative modality, such as IMRT. Both of these strategies keep practitioners from using proton technology optimally. Range modulation has been deployed across a number of different tumor types for various planning scenarios since August 2010 when first developed. In order to make this comparison valid, one patient was selected and three plans were constructed to demonstrate the use of this new technique.

## Methods

**Figure 1:**
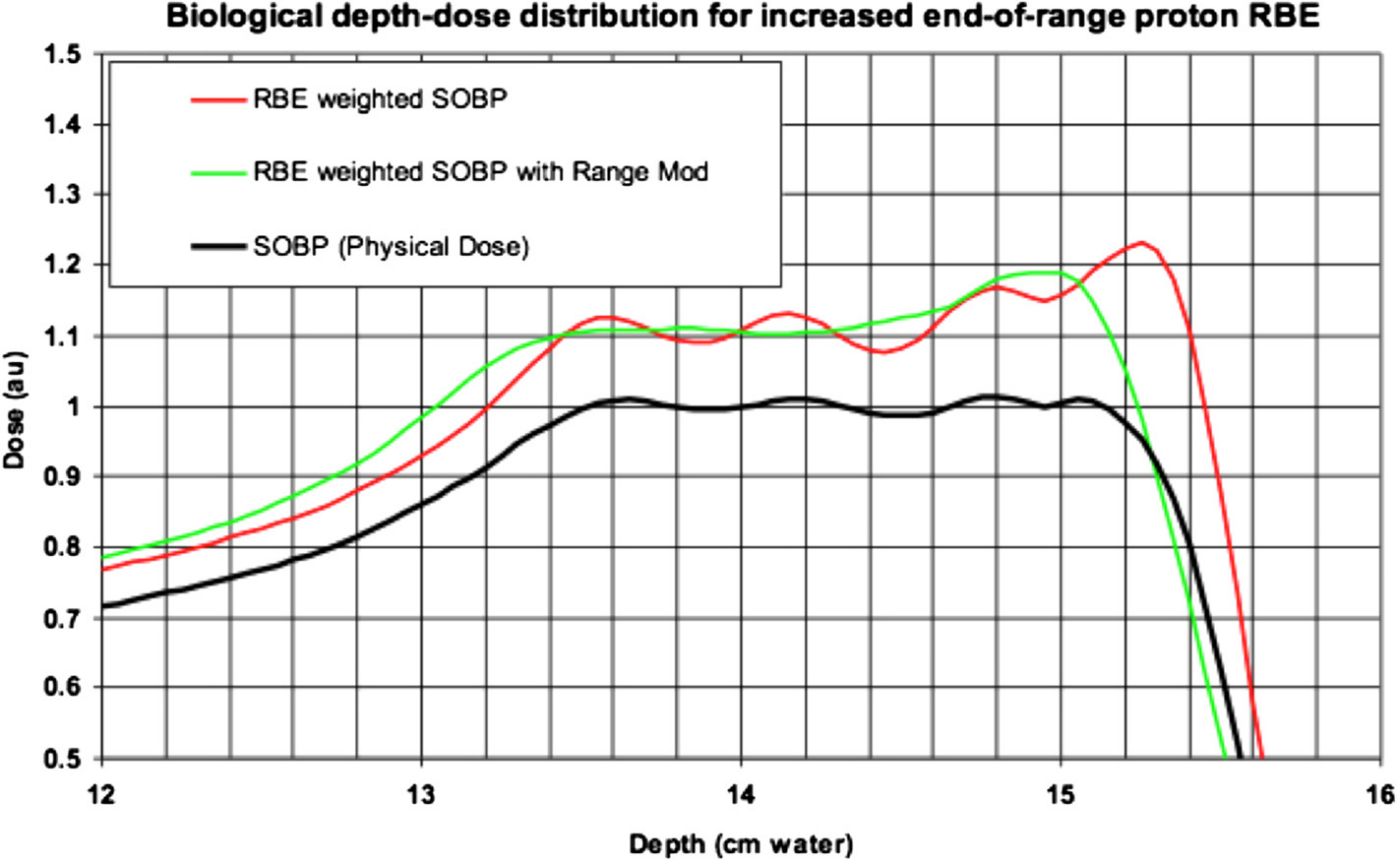
**The physical dose for a SOBP composed of four pristine Bragg peaks each separate by 6 mm water equivalent.** Applying our illustrative model of increased distal RBE to the individual pristine peaks produces the RBE weighted SOBP. The “range mod” technique mitigates the changes in SOBP plateau flatness, range, and effective dose at the distal edge. Here the modulation is achieved by splitting the SOBP into two parts and shifting one by 3 mm to both smooth out the SOBP and decrease the RBE at the end of the beam.

**Figure 2:**
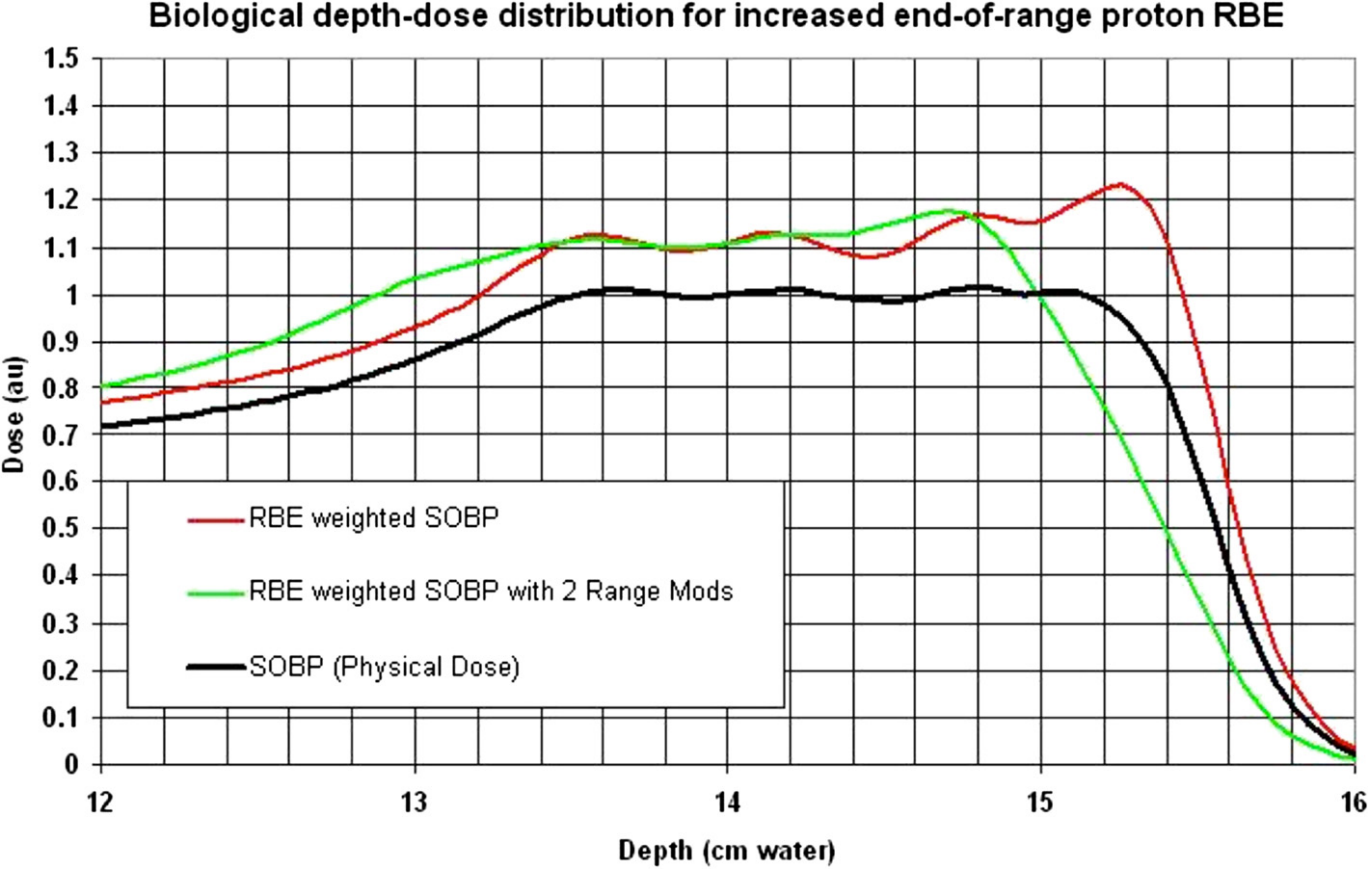
**Splitting the SOBP into three beams so as to further reduce the RBE effect.** This what is typically done when a single beam plan is being used such as with a posterior fossa boost or germinoma boost after whole ventricular radiation is employed. It can be employed at other times as well when there is significant clinical concern regarding a specific organ at risk.

This manuscript describes a novel technique to mitigate issues related to increased RBE at the distal edge of the SOBP by spoiling the distal falloff with existing patient specific device (PSD) sets and beam angles. We call this technique “range modulation” or “range mod”. This is accomplished by splitting the dose planned for a beam in half, shown in Figure 1, and then delivering half the dose as planned and other half of the dose with an identical beam whose range has been modified by 3 mm [see reference on treatment system ref [13], 3 mm is half the 6 mm spacing between pristine peaks in the SOBP for our beam delivery system and comparable to the potential 1–2 mm increase in range due to RBE). If a single beam is being used for a plan to a significant dose, three beams are used and the range is modified by 2 mm for each beam making the two beam range changes 2 mm and 4 mm as shown in Figure 2. With this technique, OARs are spared unnecessary dose. As an example, we assumed the proton beam to have a uniform RBE of 1.1 and modeled the excess RBE analytically with a hyperbolic tangent centered on the point of maximum dose of the pristine Bragg peak and saturating at 35% with a characteristic length of 2 mm [8]. Applying this model to the individual pristine peaks comprising the delivery of a SOBP allows us to illustrate the changes in plateau flatness, range, and biological effectiveness -- a “range mod” mitigates all three of these effects. With this method, both PSD number and patient set up time potentially decrease. All work presented was conducted in compliance with institutional norms and doses delivered and volumes treated were within the standard of care for the case described.

The “range mod” technique will be illustrated with evaluation of a retreatment patient plan treated at our center with PBT. This patient had recurrent ependymoma in the posterior fossa. Treatment plans were constructed using 3DCRT, IMRT, NRMPBT, and RMPBT techniques. The range modulation plan employed end of range modification of 3 mm, thus avoiding complete transmission through any OAR. While not the case in the given example, our policy as noted above for plans using a single beam is to employ three separate fields, each with a unique range. In the plans shown, two range-modulated fields per beam angle were used.

Xio 6.0 treatment planning software (Elekta AB, Sweden) was used for all cases presented for PBT planning. Active scanning [17,18] as described previously was employed for the delivery of the spread out Bragg peaks (SOBPs) using apertures and compensators manufactured by IU Health Cyclotron Operations (IUCO). Our uniform active scanning process requires the use of apertures to shape the beam edge and compensators to shape the beam end via direct range compensation, as a sum these pieces of equipment are called patient specific devices (PSDs). Aperture devices were machined out of medical grade brass while compensators were machined from medical grade Lucite using standard procedures; U.S. Food and Drug Administration (FDA) requisite quality assurance was performed. All beam outputs and devices were checked for accuracy before treatment delivery per routine. At our center, each treatment position’s verification images are reviewed by a physician for every fraction in real time either at the gantry or via remote viewing monitor prior to delivering beam.

The DICOM RT data set from the patient’s plan computed on XiO was recovered and imported in the Eclipse 10 (Varian Medical Systems, USA) for side-by-side comparison use. All photon plans were constructed within Eclipse 10. The deployed plan was compared to chart data prior to de-identification in order to confirm the correct recovery of the data and then doses to all contoured structures were converted into percentage format for comparison.

The patient had been previously treated to 54 Gy via coplanar IMRT and relapsed in field. This patient received RMPBT at our center in order to avoid OARs approximately two years ago and is currently doing well without evidence of radiation damage or other local toxicity.

## Results and discussion

**Figure 3:**
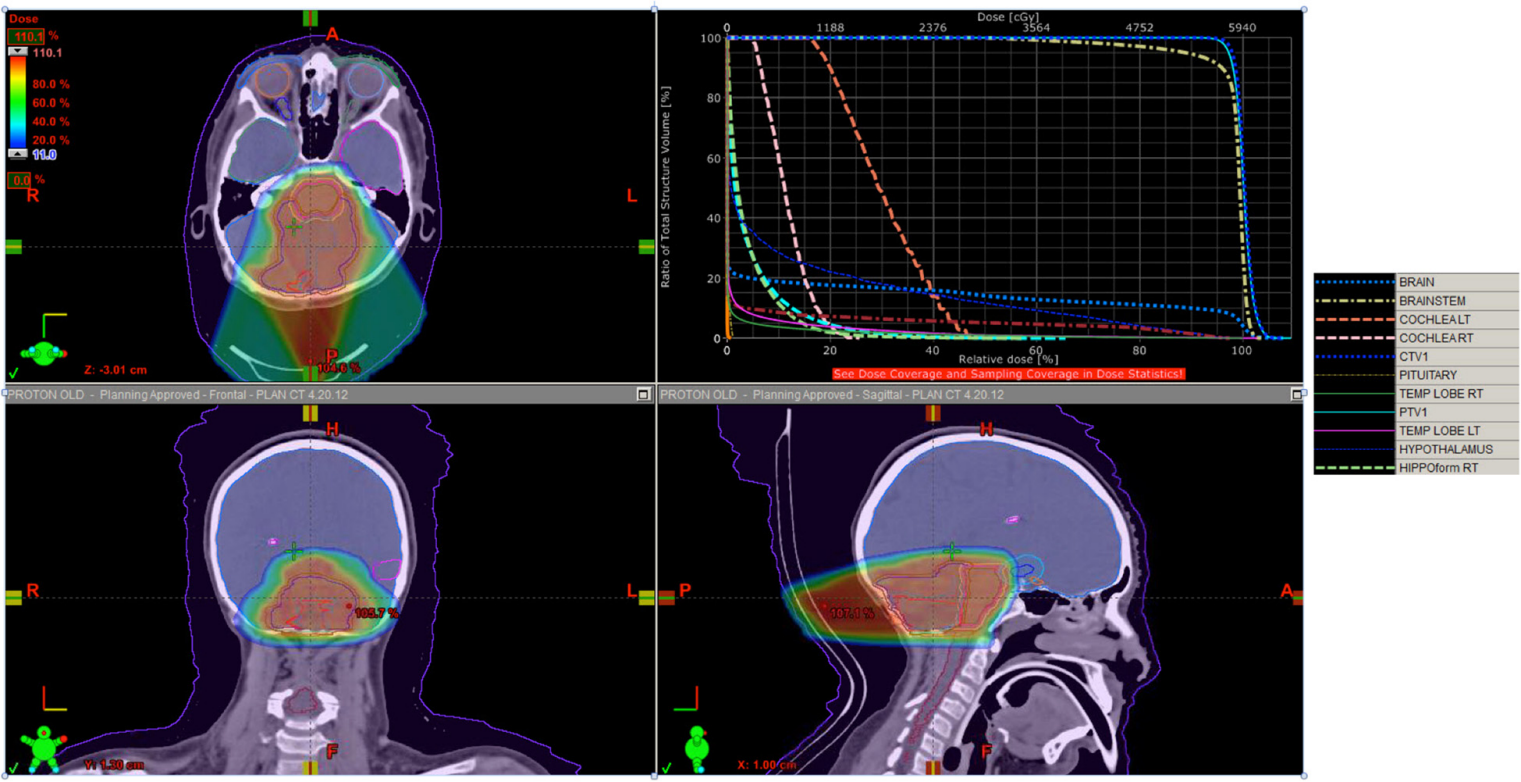
The plan using NRMPBT.

**Figure 4:**
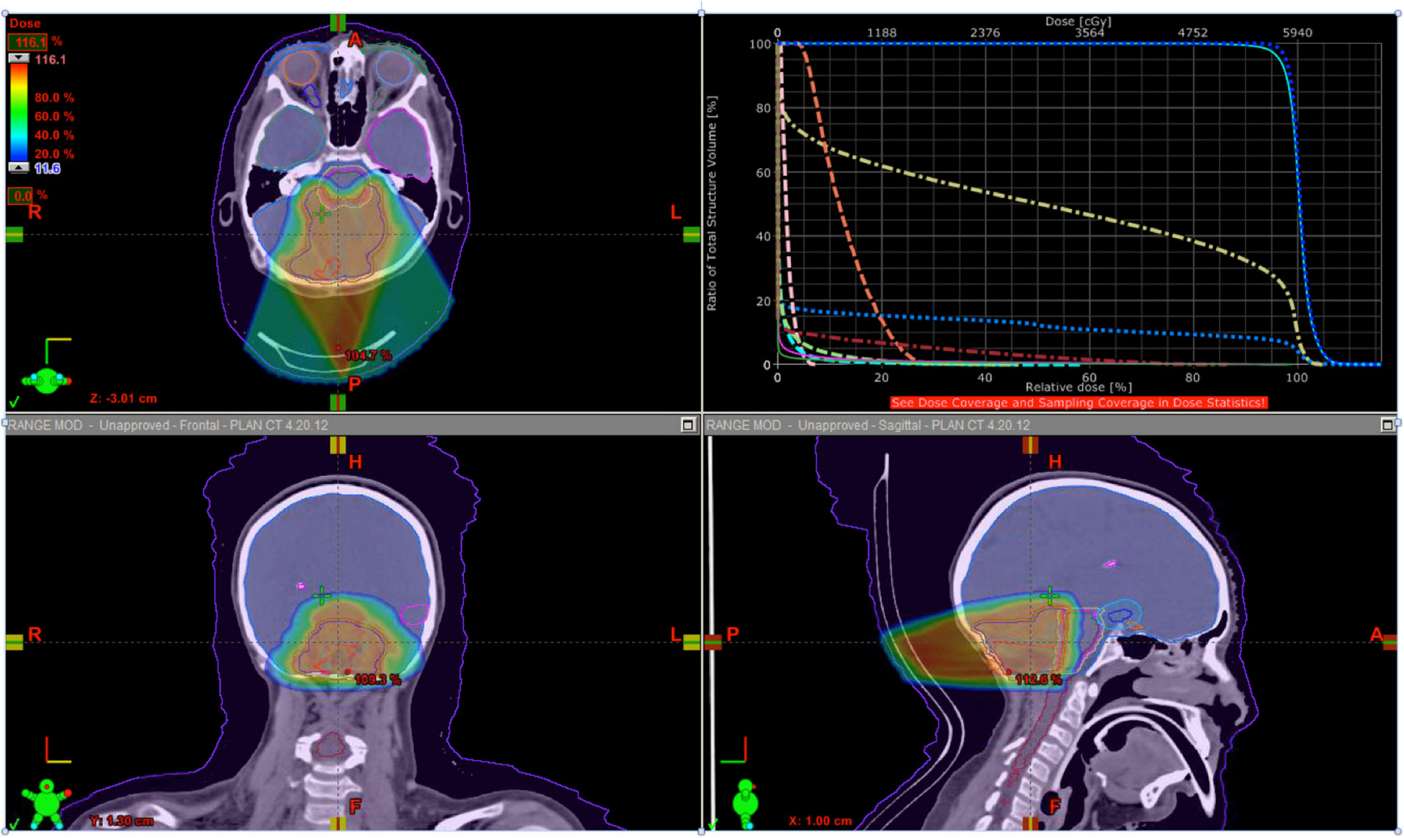
**The plan using RMPBT as the primary treatment. DVH colors are the same as used in Figure **3**.**

**Figure 5:**
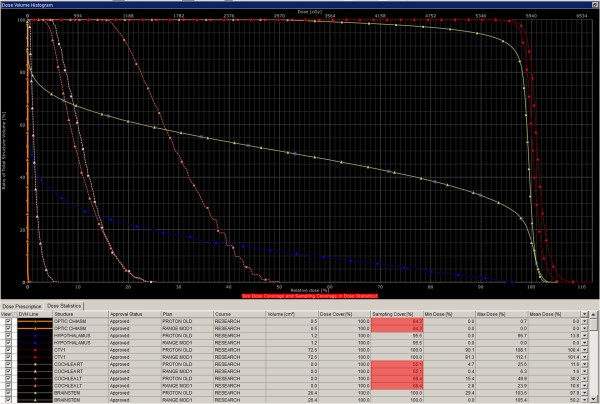
**Comparison of the DVH’s for several OAR’s between the NRMPBT and the RMPBT plans shown in Figures **3**and**4**respecitively.** In every case the RMPBT plan treats less volume of the OAR’s shown.

**Figure 6:**
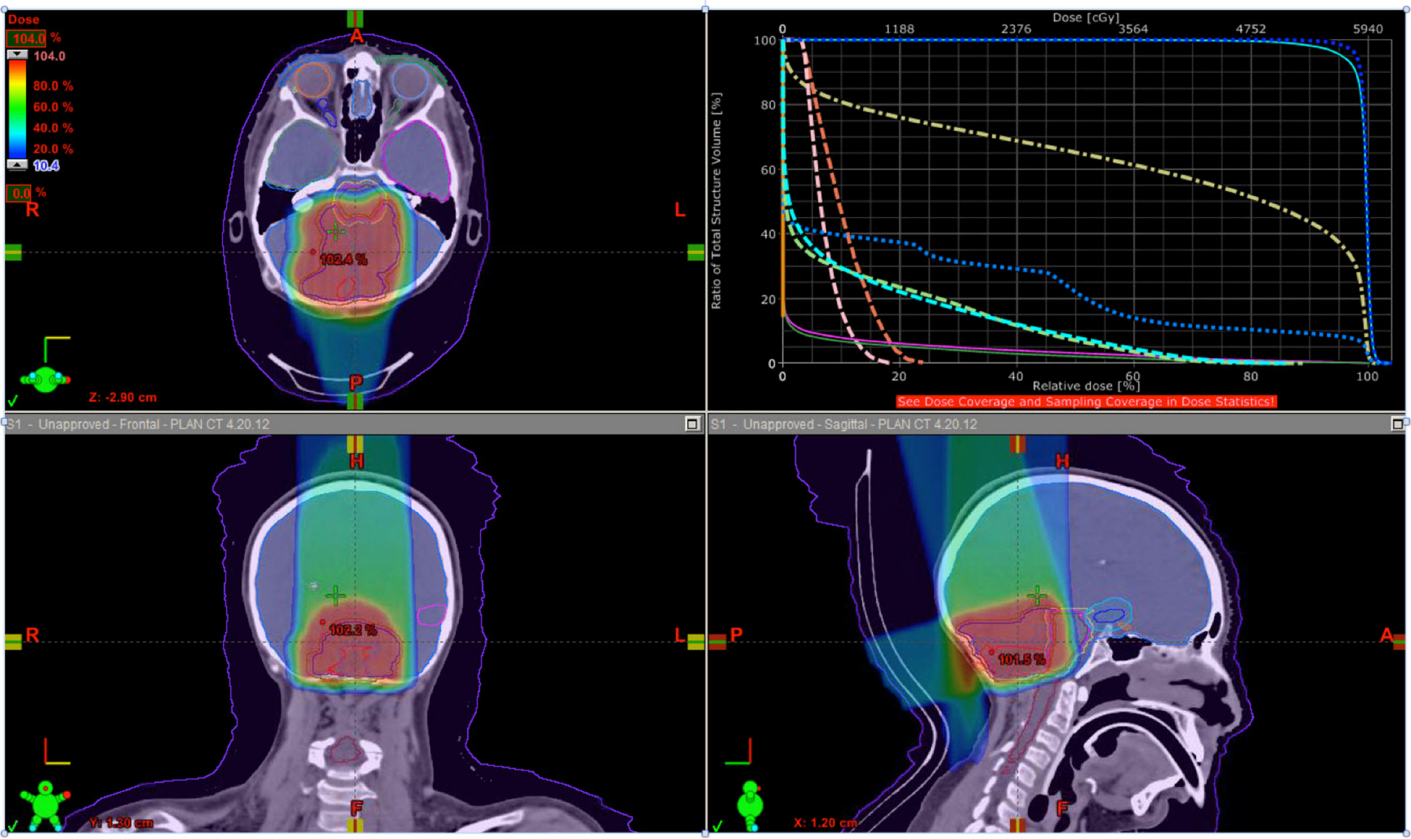
**The actual plan delivered using RMPBTrt (as part of retreatment).** Vertex beams are used to minimize dose summation with the prior coplanar IMRT plan this patient received. DVH colors are the same as used in Figure 3.

Multiple beam plans were generated as primary treatment for this patient using NRMPBT (Figure 3), RMBPT (Figure 4), and a comparison of DVH’s for the NRMPBT and RMPBT plans (Figure 5) are shown. Additionally, the actual retreatment plan is shown in order to illustrate how RMPBT is used in this special context and is labeled RMPBTrt (Figure 6). It varies from the optimal RMPBT plan in that a vertex field is used so as to pull dose off the skin and minimize the volume of retreated tissue outside of the PTV. Table 1 outlines the dosimetric comparison of the plans.

**Table 1:** Dosimetric comparison of three plans

		**CTV**	**PTV**	**Brainstem**	**Left cochlea**	**Right cochlea**	**Left temporal lobe**	**Right temporal lobe**	**Hypothalamus**	**Pituitary**	**Left hippocampus**	**Right hippocampus**	**Brain**
NRMPBT	Min	90.1	88.7	29.4	15.4	4.7	0	0	0	0	0	0	0
	Max	108.1	109.4	103.6	49.9	25.6	102.8	100	96.7	1.2	65.8	57.8	108.5
	Mean	100.4	100.1	97.9	30.2	11.6	2.2	1.5	13.8	0.2	4.4	4.1	14.4
RMPBT	Min	79.9	63.2	0	3.9	0	0	0	0	0	0	0	0
	Max	110.1	110.1	105.8	29.2	7.4	97.9	99.1	0	0	58.8	46.6	110.1
	Mean	100.7	100.3	50.1	12.9	1.9	0.9	0.6	0	0	0.8	1.1	12.4
RMPBTrt	Min	55.5	45.6	0	8.1	4.4	0	0	0	0	0	0	0
	Max	100	100	96.7	27.7	15.2	94.8	92.5	0	0	81	70.3	100
	Mean	94.5	93.6	42.4	16.9	8.2	4.3	2.6	0	0	11.5	8.7	20.7

All numbers are in percentage of prescription dose. The prescription dose for this case was 59.4 Gy.

All planning modalities produced plans that cover the PTV. The RMPBT method treats less total brain than the NRMPBT method given the fact that the beams are not extended to cover the entirety of the brainstem in an effort to avoid ending the beam in the brainstem. When looking at the OAR doses, the difference in plans is pronounced due to this difference. Data exist that suggest doses over 10 Gy are sufficient to ultimately cause hypothalamic dysfunction [19]. In the NRMPBT plan, the average dose is lower, but the peak dose posteriorly is close to the full prescription dose due to the goal of treating through the full brainstem. Only in the RMPBT plan is hypothalamic dose absent completely. This trend continues for the doses to left and right cochleae, the temporal lobes, the pituitary, and the brainstem itself. These data are summarized in Table 1.

As a formal retreatment plan, behind the numbers in the RMPBTrt plan is the concept of treating the previously treated tissue to the lowest sum doses possible. This was achieved by minimizing dose overlap issues between a prior co-planar photon plan and the current retreatment proton plan (shown) via vertex beam usage. The through brainstem approach or NRMPBT plan, had a vertex field been employed, treats a much larger volume of hippocampus and temporal lobe, making its use problematic in the retreatment context.

Even with the RMPBTrt plan’s beam arrangement used to minimize overlap with prior dose, very significant dosimetric saving occurred for the cochleae, the hypothalamus, and the brainstem relative to the NRMPBT plan. The hippocampal dose, despite the vertex field, remained well below the mean dose seen by the other methods.

The value of RMPBT is one of significantly increased patient safety by the direct reduction of treated tissue in a fashion otherwise impossible even for traditional proton therapy because it allows the safe termination of a proton beam or set of beams in an OAR. The technique can be adapted to use in pencil beam planning as well and may prove to be even more critical in that arena as beam edge dosimetry will likely need to become modulated as well.

In each case the number of PSD sets used was decreased or kept the same. We hypothesize that time in the room and complexity was decreased in every range modulated patient scenario relative to electing another angle from which to treat. Range modulation was simple to deliver in the treatment rooms. Finally, as a result of fewer net patient positions being used, fewer verifications films are needed and patient exposure to radiation was decreased.

There are four main advantages to range modulation, or smearing of the distal range of a proton beam, compared to traditional multiple beam proton therapy:

1. Better tissue sparing is achieved via a more aggressive use of distal blocking.

2. Theoretical time-savings in the treatment room as fewer beam angles are needs. This could allow the avoidance of anesthesia in some cases. It also decreases the need to wait for the physicians required to review position films (every field is reviewed every day in our center prior to beam delivery), improving throughput.

3. Less image guidance imaging is used as only the first of a range modified series of beams required image guidance (orthogonal image verification).

4. No new PSD sets have to be manufactured which saves time for the machine shop construction and the cost of the materials and labor involved.

Range modulation as a methodology is not able to solve all problems and can introduce new problems into a plan. There is still entry dose overlap, it adds to the complexity of a plan by adding more beams to a plan, and if too few beams are used skin tolerance can be an issue. Beam angle variation also can be quite valuable to make plans more robust as target and other tissue volumes change during treatment. This is important in anatomical regions containing tissue/air/bone such as the sinuses and hilar regions. This method is a new way of employing the primary principle of radiation safety of “as low as reasonably achievable,” more commonly known by the acronym ALARA, in treatment. It is cost-effective because no new PSD sets need to be constructed and the patient beam angle does not change in the room taking time and requiring set-up imaging for position verification. The presentation of this method is obviously limited by the presentation of only one case, but the idea has translated in our clinic to spinal cord cases, craniopharyngiomas, optic pathway tumors, base of skull tumors, and pelvic tumors. Careful evaluation of the method will demonstrate that the method succeeds by moderately smearing out the sharpness of the end of the SOBP. This modest compromise allows safe stoppage of proton beams within critical structures such as the brainstem as shown. Ultimately it will be up to the treating physician to balance the need for safety against distal blocking goals regarding whether a biologic hot spot in an OAR in a given plan is acceptable.

The method presented in this paper confronts a clinical problem inherent in charged particle therapy – the safe and effective management of the increasing RBE at the end of particle beams. In all forms of past, present, and future of charged particle therapy, the use of this method or an analogous approach will be of use to the clinician when there is an RBE increase at the end of the beam being used. One could even expand this idea to any non-linearity found in RBE in beams as scanned beams allow physicians to compensate for these issues. Our future particle therapy treatment planning will likely also be RBE focused rather than solely energy focused. Table 2 summarizes the method’s usage in our clinic and represents general guidelines.

**Table 2:** Informal range modulation guidelines employed in our clinic

**Clinical situation**	**Approach used**	**Example**
**Single beam being employed for more than a few fractions.**	**Three ranges rather than two are used.**	**1. Full posterior fossa boost with full cochlear sparing.**
		**2. Boost for germinoma after whole ventricular radiation often via a posterior beam.**
**Three or more main angles are being used and the patient is awake meaing six possible fields may need to be delivered.**	**One of two ranges for each beam angle is treated per day with care to avoid coincidental beam ends. Ranges alternate each day.**	**1. Brain tumors.**
		**2. Pelvic tumors.**
		**3. Spine tumors in some cases.**
		**Base of skull tumors.**
**The patient has had prior radiation.**	**We will sometimes use three ranges rather than two when super critical structures are involved.**	**1. Ependymoma retreatment with the brainstem.**
		**2. Salvage glioma cases with beams ending in eloquent brain.**
		**3. Retreatment patients with a distant history of radiation necrosis with new cancer in similar locations.**
**Two or more beams end in the same point or points.**	**Beams are split into range mod pairs and care is used to look at each end point set for each day to avoid overlaps.**	**1. Fourth ventricular ependymoma.**
		**2. Vertex beams use can hide this issue and great care is used in plan review to look for “in corner” doses.**

## Conclusions

This present study illustrates a novel method that mitigates the increased RBE at the end of the SOBP in proton treatment planning. It may not be applicable in all situations and decreases the sharpness of the dose fall-off at the end of the SOBP as a result. It has proven practical to design and implement in our clinic. It is most often used in plans using multiple beams. The method represents treatment planning that reflects not only thinking in terms of traditional energy dose (Gy) but also in terms of biologic dose (RBE).

## Abbreviations

RBE: Relative biological effectivess; SOBP: Spread out Bragg peak; PBT: Proton beam therapy; AU: Srbitrary units; 3DCRT: Three dimensional conformal radiation therapy; IMRT: Intensity modulated radiation therapy; NRMPBT: Non-range modulation proton beam therapy; RMPBT: Range modulation proton beam therapy; RMPBTrt: Range modulation proton beam therapy (re-treatment scenario); OAR: Organ at risk; Gy: Gray; PSD: Patient specific device; FDA: U.S. Food and Drug Agency; PTV: Planning target volume; CTV: Clinical target volume.

## Competing interests

The authors declare that they have no competing interests.

## Authors’ contributions

JB invented the idea behind this method, set up its use in the clinic, and was the primary individual behind the interpretation of the plans for patients treated in this fashion. He drafted the paper and served as the main author of the paper. MMcM made substantive intellectual contributions to the idea in this paper and helped draft the paper. PJ made substantive intellectual contributions to the idea in this paper and helped draft the paper. TH constructed the first plans using the method and did the proton therapy plans in this paper. MM contributed to the paper early in the idea formation process via critical discussions with JB. CWC made intellectual contributions to the idea in this paper and helped draft the paper. ID made intellectual contributions to the idea in this paper and helped draft the paper. KM made intellectual contributions to this paper and helped draft the paper. MW made substantive intellectual contributions to the idea in this paper and helped significantly in the drafting of the paper. All authors read and approved the final manuscript.

## Authors’ information

Each author is associated with the IU Health Proton Therapy Center and the IU School of Medicine. Please see http://iuhealth.org/proton-therapy-center/ and http://radonc.medicine.iu.edu for further information.
